# Ready vascular permeability of a near-infrared fluorescent agent ASP5354 for intraoperative ureteral identification enables imaging of carcinoma tissues

**DOI:** 10.1038/s41598-023-37025-z

**Published:** 2023-06-17

**Authors:** Katsunori Teranishi

**Affiliations:** Graduate School of Bioresources, Mie University, 1577 Kurimamachiya, Tsu, Mie 514-8507 Japan

**Keywords:** Biological techniques, Cancer, Optics and photonics

## Abstract

This study investigates the ability of a near-infrared fluorescence (NIRF) imaging agent, ASP5354, for in vivo fluorescence imaging of esophageal squamous cell carcinoma (ESCC) tissues. The ability of ASP5354 was evaluated using a single dose of ASP5354 or indocyanine green (ICG), which was intravenously administered to a KYSE850 human ESCC xenograft mouse model. Subsequently, in vivo NIRF images of the mouse were obtained using a clinically available camera system. ASP5354-specific NIRF signals were strongly detectable in KYSE850 carcinoma tissues immediately (30 s) following ASP5354 administration compared with normal tissues. Meanwhile, ICG could not distinguish between normal and carcinomatous tissues. To elucidate the associated imaging mechanisms, the vascular permeability of ASP5354 and ICG was investigated in rat back dermis treated with saline or histamine, which enhances vascular permeability, using in vivo NIRF imaging. ASP5354 exhibited higher vascular permeability in histamine-treated skin than in normal skin. KYSE850 carcinoma tissues can be distinguished from normal tissues based on the measurement of ASP5354-specific NIRF signals, and the mechanism that enables imaging relies on the specific and rapid leakage of ASP5354 from the capillaries into the stroma of carcinoma tissues.

## Introduction

Near-infrared fluorescent indocyanine green (ICG) is a green dye that was approved by the Food and Drug Administration (FDA) as a diagnostic agent for liver function in 1959 and has since been widely used. ICG emits near-infrared fluorescence (NIRF) at approximately *λ*_max_ 800 nm and has recently been used in NIRF image-guided surgery, including lymph node identification and blood vessel imaging^1,2^. ICG is specifically and rapidly transferred to the liver after intravenous administration and is subsequently excreted into the bile juice. However, cancer tissues in the liver excrete ICG more slowly than normal tissues and can be intraoperatively identified using the NIRF of ICG^3,4^. A near-infrared fluorescent agent, ASP5354 (emission *λ*_max_ 815 nm in whole blood, Suppl. Fig. 1) was developed for intraoperative ureter identification during abdominal surgery^5–9^. The safety, tolerability, and pharmacokinetics of ASP5354 were assessed in a phase 1 study^9^, and a phase 3 study is currently underway^10^. ASP5354 comprises two *β*-cyclodextrin molecules that are covalently bound to a heptamethine indocyanine molecule, which forms the skeleton of the ICG molecule; the hydrophobic heptamethine indocyanine moieties are coated with *β*-cyclodextrin moieties, which can include hydrophobic molecules in the cyclodextrin cavity^11^. This structural property facilitates specific and super-rapid renal excretion following intravenous ASP5354 injection, resulting in the emission of strong NIRF from ASP5354 in urine within the ureters.

To expand the in vivo application of ICG for cancer imaging, the potential of ASP5354 has been assessed using the Renca mouse carcinoma orthotopic mouse model^12^, MB49 mouse bladder cancer orthotopic mouse model^13^, and MKN-45 human gastric cancer xenograft mouse model^14^. In the MKN-45 cancer imaging study, although MKN-45 cells failed to take up ASP5354 from a 2.4-μmol/L solution, ASP5354 emitted stronger NIRF in MKN-45 cancer tissues than normal tissues immediately following intravenous injection of ASP5354 (single 120 nmol/kg body weight dose) to MKN-45 cancer xenograft mice^14^. However, the mechanisms underlying the high NIRF of ASP5354 in cancer tissues remain unclear.

In 2020, 604,100 cases (ranked seventh among all carcinomas) of esophageal carcinoma were diagnosed worldwide, with 544,076 reported esophageal carcinoma–related deaths (ranked sixth among all carcinomas)^15^. The two dominant histological subtypes of esophageal carcinoma are esophageal squamous cell carcinoma (ESCC) and adenocarcinoma. Early detection and treatment of esophageal carcinoma can improve patient prognosis and survival rates. In early-stage ESCC, changes in the mucosa are subtle and are often undetected during endoscopic examinations using white light. To increase sensitivity, Lugol chromoendoscopy using Lugol's stain (iodine–potassium iodide solution) and white light is currently the gold standard for ESCC diagnosis^16^. Normal esophageal mucosa stains brown after spraying with Lugol's solution, whereas ESCC mucosa does not. However, Lugol’s solution can induce iodine hypersensitivity and esophagitis^17^, thus limiting its application. Meanwhile, although various fluorescent contrast agents have been reported for in vivo targeted imaging^18–21^, including a fluorescent glucose–labeled agent, 2-DG800CW, and a piperazine-coumarin-based fluorescent agent^22,23^, no such agents have demonstrated clinical efficacy for distinguishing ESCC.

The current study seeks to assess the potential of ASP5354 as a contrast agent for NIRF differentiation between ESCC and normal tissues using a KYSE850 cell (human ESCC line^24^) xenograft mouse model. Moreover, the mechanism underlying the ability of ASP5354 to differentiate between carcinoma tissues, including ESCC and MKN-45, and normal tissues is investigated.

## Results

### In vivo NIRF imaging of KYSE850 carcinoma in the subcutaneous xenograft mouse model

**Figure 1:**
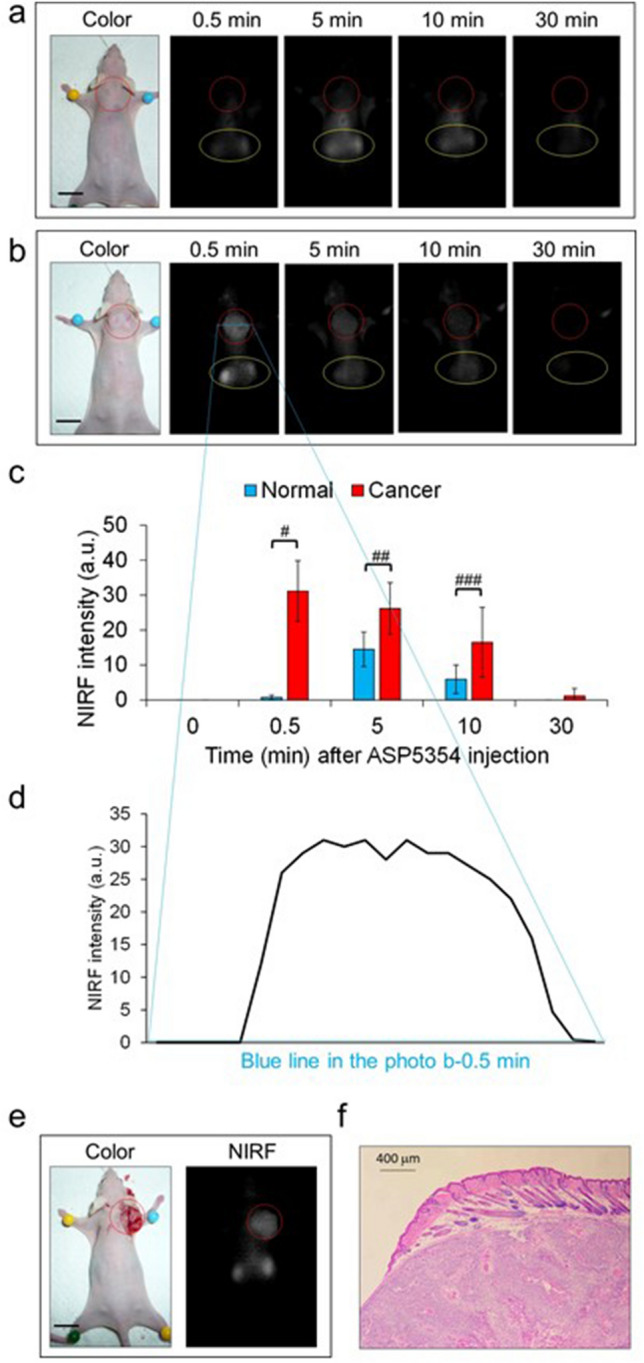
Near-infrared fluorescence (NIRF) of mouse back tissues bearing KYSE850 human esophageal squamous cell carcinoma following intravenous administration of ASP5354. The NIRF images in (**a**) and (**b**) were obtained under the same measurement conditions at a excitation level (4 a.u.) of camera system. NIRF appears white. Yellow ellipses denote NIRF derived from the kidneys. (**a**) NIRF images in the back of a representative normal mouse injected with vehicle (red circles). Scale bar: 10 mm. (**b**) NIRF images in the back of a representative mouse bearing KYSE850 carcinoma (red circles). Scale bar: 10 mm. (**c**) Mean ± SD of NIRF intensities in center of carcinoma (*n* = 7) and normal tissues (*n* = 3) in 5 mm × 5 mm square. ^#^*p* < 0.0005, ^##^*p* < 0.05, ^###^*p* < 0.1. (**d**) NIRF intensity on the blue horizontal axis in the image at 0.5 min in (**b**). (**e**) NIRF in incised KYSE850 carcinoma tissue (red circles) at 1 min after ASP5354 injection. Scale bar: 10 mm. (**f**) Histopathological analysis of carcinoma tissues harvested from KYSE850 carcinoma skin 1 min after intravenous administration of ASP5354.

Vehicle control mice and mice with KYSE850 carcinoma xenografts in the subcutaneous space were imaged at a moderate excitation level (4 a.u.) of camera system before (at time zero) and after intravenous administration of a single ASP5354 dose of 120 nmol/kg body weight. The in vivo NIRF signals in normal and tumor were not detected at time zero. The in vivo NIRF signal of normal tissue following administration of the vehicle control was relatively low (NIRF intensity: 0.75 ± 0.64 [mean ± SD] a.u.) at 0.5 min after ASP5354 injection. Five minutes after ASP5354 injection, the NIRF signal was detected along the entire back, peaking in the neck peaked and subsequently decreasing (Fig. 1a,c). A clear in vivo NIRF signal of ASP5354 associated with implanted KYSE850 carcinoma cells in the subcutaneous spaces was detected (NIRF intensity: 31 ± 8.7 [mean ± SD] a.u.) 0.5 min after ASP5354 injection, and the NIRF signal decreased 5 min after ASP5354 injection (Fig. 1b,c). The NIRF intensity ratios of carcinoma/normal tissues were 41 at 0.5 min, 1.8 at 5 min, and 2.8 at 10 min after ASP5354 injection. Meanwhile, 30 min after ASP5354 injection, the NIRF signals in the carcinoma regions were consistently stronger than those in the normal tissues of the vehicle controls (Fig. 1c). By 0.5 min, the difference in NIRF intensity at the boundary between the carcinoma and normal tissues in Fig. 1b was clear (Fig. 1d), indicating that ASP-5354 could distinguish between carcinoma and normal tissues. Additionally, the NIRF signal of ASP5354 in the kidneys penetrated the skin of the back (Fig. 1a,b).

One minute after intravenous injection of ASP5354 into KYSE850 carcinoma xenograft mice, the carcinoma tissue was incised and NIRF in the carcinoma tissue was measured. An NIRF signal intensity of 49 a.u. (*n* = 1) was observed, indicating that NIRF could be used to distinguish carcinomas from normal tissues (Fig. 1e). Carcinoma and skin tissues were dissected, frozen, and sectioned. Frozen sections of adjacent tissues were also stained with hematoxylin and eosin (H&E) and observed under a microscope (Fig. 1f). NIRF imaging of frozen sections was performed using an NIRF microscope. No NIRF signal was detected, even under high-sensitivity microscopic conditions (photo not shown).

**Figure 2:**
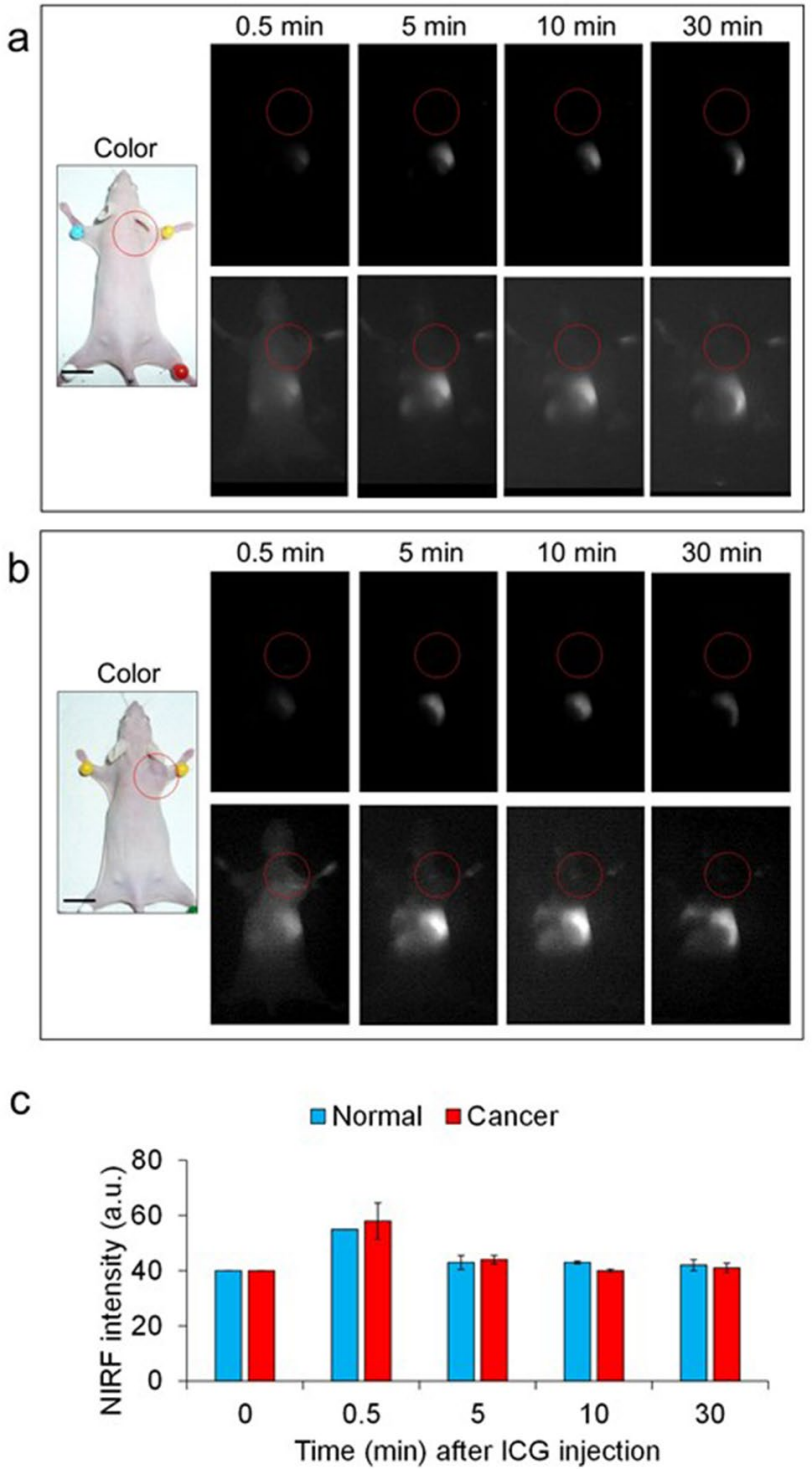
NIRF in the back tissues of mice bearing KYSE850 human esophageal squamous cell carcinoma after intravenous administration of indocyanine green (ICG). Upper NIRF images in (**a**) and (**b**) were obtained under the same measurement conditions as those in Fig. 1a,b. Lower NIRF images in (**a**) and (**b**) were obtained at higher excitation level (8 a.u.) of camera system than the upper-panel images. NIRF appears white. Scale bar: 10 mm. (**a**) NIRF images in the back of a representative normal mouse injected with vehicle (red circles). (**b**) NIRF images in the back of a representative mouse bearing KYSE850 carcinoma (red circles). (**c**) Mean ± SD of NIRF intensities in the center of carcinoma (*n* = 3) and normal tissues (*n* = 3) in a 5 mm × 5 mm square in NIFR images obtained at excitation level 8 a.u.

Before (at time zero) and after intravenous administration of a single dose of ICG at 120 nmol/kg body weight via the tail to normal and KYSE850 xenograft mice, in vivo NIRF signals were imaged at a moderate excitation level (4 a.u.) of camera system. After the administration, NIRF was detected along the entire back, particularly in the middle and lower regions of the back, but not in tumor tissues (upper photos in Fig. 2a,b). When the imaging was performed at a high excitation level (8 a.u.), background noise appeared throughout the images independent on body before ICG injection. Even with the high sensitivity provided by the NIRF camera system, no tumor-specific NIRF signals of ICG was detected in the carcinoma tissue 30 min after ICG injection (lower photos in Fig. 2a–c).

### In vitro ASP5354 uptake by KYSE850 cells

The uptake of ASP5354 by KYSE850 cells was also evaluated. KYSE850 cells were incubated with 2.4-µmol/L ASP5354/PBS for 10 min. After washing with free ASP5354, the cells were subjected to microscopic NIRF imaging. No significant uptake of 2.4-µmol/L ASP5354 was observed (Suppl. Fig. 2).

### Vascular permeability of ASP5354

**Figure 3:**
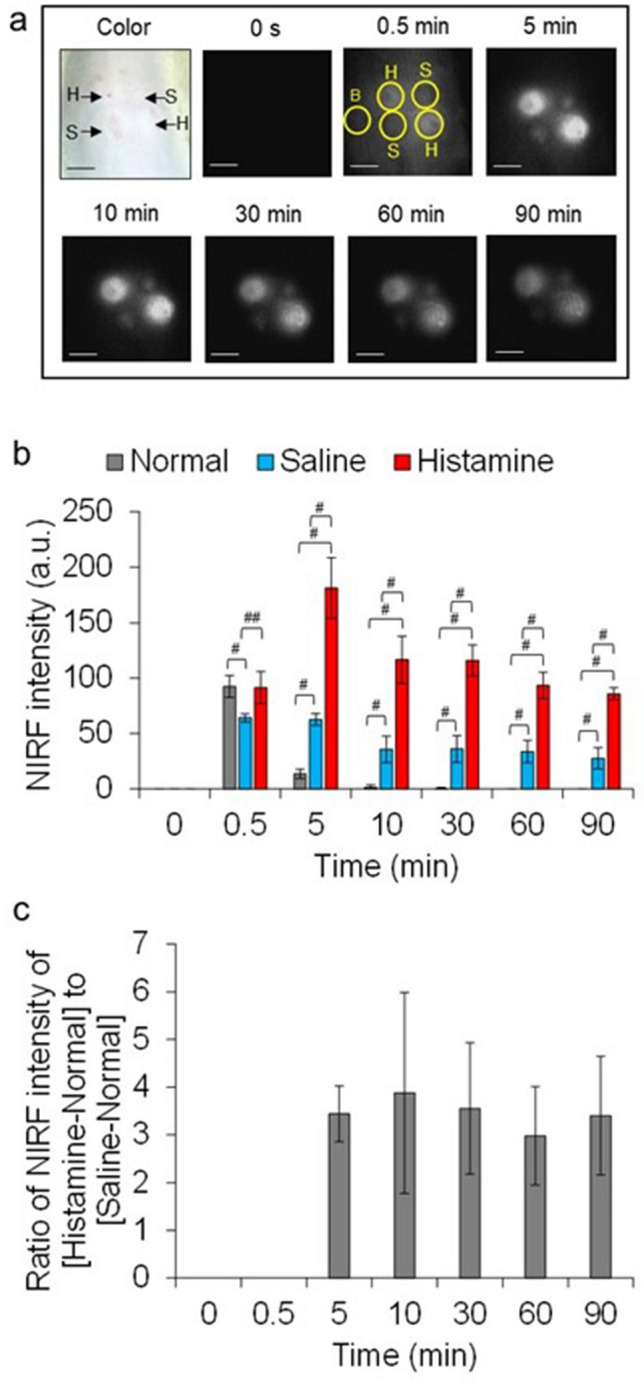
Vascular permeability of indocyanine green (ICG) in rat back tissues. (**a**) Histamine (4.5 mmol/L in saline, 0.05 mL per site for 300 g rats) or saline (0.05 mL per site for 300 g rats) was injected in the back dermis. Immediately ICG (250 nmol/kg body weight) was intravenously injected in tails under measurement of near-infrared fluorescence (NIRF) over 90 min. B: normal site, H: histamine-treated site, S: saline-treated site. NIRF appears white. Scale bar: 10 mm. (**b**) Mean ± SD of NIRF intensities in circles in the photos of (**a**) (*n* = 4). ^#^*p* < 0.005, ^##^*p* < 0.05. (**c**) Mean ± SD of ratio of NIRF intensities (*n* = 4).

**Figure 4:**
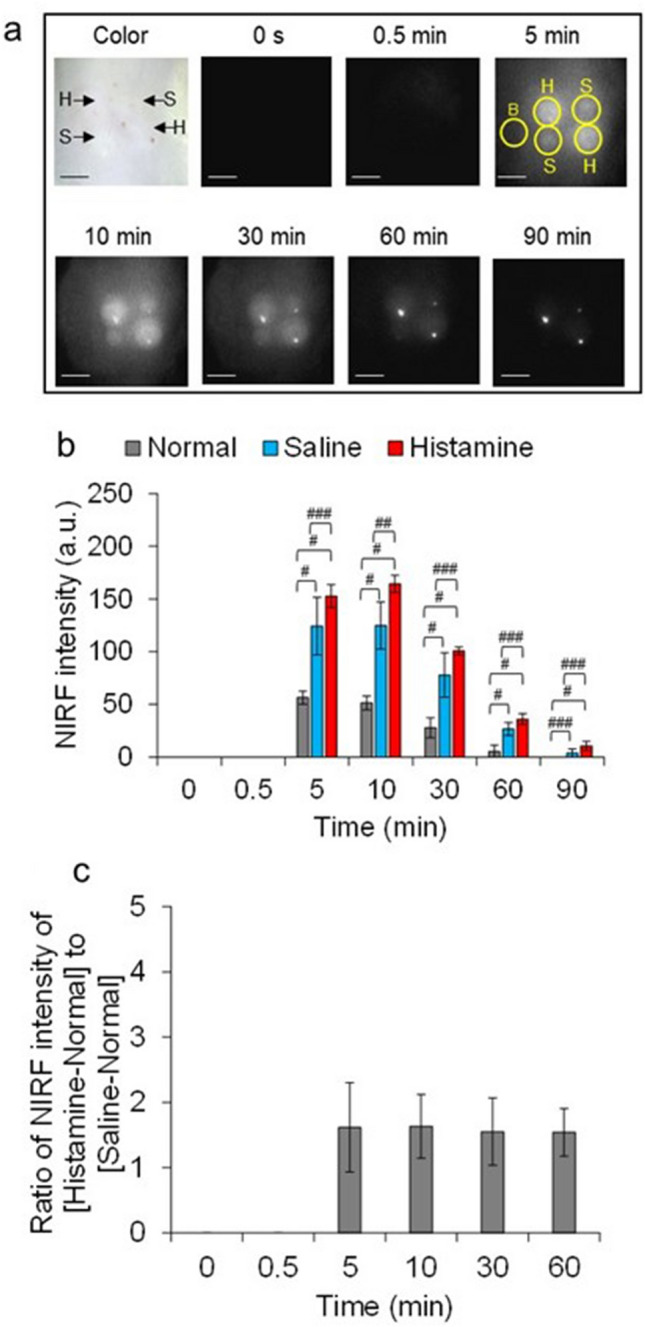
Vascular permeability of ASP5354 in rat back tissues. (**a**) Histamine (4.5 mmol/L in saline, 0.05 mL per site for 300 g rats) or saline (0.05 mL per site for 300 g rats) was injected in the back dermis. Immediately ASP5354 (250 nmol/kg body weight) was intravenously injected in tails under measurement of near-infrared fluorescence (NIRF) over 90 min. B: normal site, H: histamine-treated site, S: saline-treated site. NIRF is displayed with white. Scale bar: 10 mm. (**b**) Mean ± SD of NIRF intensities in circles in the photos of (**a**) (*n* = 4). ^#^*p* < 0.005, ^##^*p* < 0.05, ^###^*p* < 0.1. (**c**) Mean ± SD of ratio of NIRF intensities (*n* = 4). Ratio at 90 min is not presented because the NIRF intensities in saline- and histamine-treated skins were too low.

The in vivo vascular permeability of ASP5354 was assessed in the back dermis of rats injected with saline or histamine, which enhances vascular permeability^25^, or left untreated, using in vivo NIRF imaging compared with ICG. NIRF intensity in the skin with non-treatment increased 0.5 min after intravenous injection of ICG, while NIRF intensity decreased 5 min after injection (Fig. 3a,b). The NIRF of ICG in skin treated with saline or histamine was greater than that in untreated skin 5 min after ICG injection with significant difference untreated skin and saline- or histamine-treated skins at *p* < 0.005. Subsequently, the NIRF intensity in the skin was relatively constant (Fig. 3a,b), and histamine treatment maintained approximately 3.5 times higher NIRF intensity than saline treatment (Fig. 3c). The NIRF intensity of ASP5354 in the untreated, saline-, or histamine-treated skin peaked 5 or 10 min after the intravenous injection of ASP5354; subsequently, the intensity gradually decreased by 90 min with significant difference untreated skin and saline- or histamine-treated skin at *p* < 0.005 (Fig. 4a,b). Additionally, 5 to 60 min after ASP5354 injection, the NIRF intensity of ASP5354 in the histamine-treated skin was consistently ~ 1.6 times higher than that in the saline-treated skin (Fig. 4c).

## Discussion

Early diagnosis and treatment of ESCC improve patient prognosis and survival. Although NIRF imaging techniques are useful for carcinoma diagnosis and intraoperative guidance, no NIRF contrast agents are currently available for clinical ESCC imaging. This study evaluated the usefulness of ASP5354 for ESCC imaging using a xenograft mouse model of KYSE850 carcinoma cells.

ASP5354 was intravenously administered and immediately emitted NIRF with sufficient ESCC-specific fluorescence intensity in KYSE850 carcinoma tissue. After ASP5354 injection, the carcinoma-to-normal (C/N) signal ratio was 41 at 0.5 min and decreased to 1.8 and 2.8 at 5 min and 10 min post-injection, respectively. The NIRF signal in the incised KYSE850 carcinoma tissue was clear and strong at 1 min compared to that in the normal tissue (C/N = 31). These results indicate that ESCC can be imaged immediately after ASP5354 injection. However, the NIRF signal of ASP5354 decreased to the NIRF level in normal tissues after 30 min, suggesting that KYSE850 carcinoma cells do not strongly take up ASP5354. Moreover, no NIRF signals were detected by microscopic examination of carcinoma tissues dissected, frozen, and sectioned 1 min after ASP5354 administration, indicating that the degree of ASP5354 uptake by carcinoma cells was exceptionally low and that the injected ASP5354 diffused away from the carcinoma tissues. Microscopic analysis of NIRF signaling in KYSE850 carcinoma cells confirmed that these cells had a low ability to uptake ASP5354. These in vivo results were consistent with in vitro examinations. Therefore, this characteristic of ASP5354 allows imaging of KYSE850 carcinoma tissues, but not cells. These results also indicate that selective uptake of imaging agents by carcinoma cells is not necessary for imaging carcinoma tissues and suggest that ASP5354 could be used clinically as an intraoperative guidance agent for imaging human ESCC tissues macroscopically for the purposes of diagnosis and directing surgery.

Considering that NIRF imaging of KYSE850 carcinoma tissues was performed immediately after intravenous administration of ASP5354, it can be administered at any time during diagnostic procedures to ensure proper imaging of carcinoma tissues, depending on the availability of patient and camera systems. Furthermore, rapid excretion of ASP5354 reduces the burden on patients. Similarly, in vivo examinations revealed that ICG did not accumulate in KYSE850 carcinoma tissues due to its rapid transport to the liver, resulting in a low NIRF intensity of ICG in the carcinoma tissue. Following injection of ASP5354 to normal mice, the NIRF intensity in the neck at 5 min was the highest among all timepoints (0.5 min, 5 min, 10 min, and 30 min). In contrast, after ICG injection to normal mice, the NIRF intensity in the neck at 0.5 min was slightly higher among the timepoints; after 5 min the intensities were the same as the background intensity (intensity at 0 min). Thus, the transport of ICG to the liver was faster than that of ASP5354 to the kidneys, and the amount of ICG inflow into the carcinoma tissues was low. Moreover, the NIRF intensity of ICG in KYSE850 carcinoma tissues did not differ from that in normal tissues immediately after intravenous injection. Therefore, the pharmacokinetic properties of ICG make it unsuitable for discriminating between the microenvironments of KYSE850 carcinoma and normal tissue stroma. The flow characteristics of ASP5354 in KYSE850 carcinoma tissues may depend on the cyclodextrin-coated structure of ASP5354 and the microenvironment of the carcinoma stroma. The characteristics also should contribute to successful in vivo imaging of MKN-45 human gastric cancer tissues in a cancer xenograft mouse model^14^.

The potential use of ASP5354 for renal cell carcinoma imaging has been previously reported^12^. In a mouse model of orthotopic renal cell carcinoma, ASP5354 was found to accumulate in normal renal tissues but not in carcinoma tissues. Meanwhile, ASP5354 flowed into MKN-45 human gastric cancer tissues^14^ and KYSE850 carcinoma tissues in a cancer xenograft mouse models more quickly than normal tissues, resulting in higher NIRF intensity the cancer tissues. Therefore, the principle of MKN-45 and KYSE850 carcinoma imaging differs from that of renal cell carcinoma imaging.

In this study, the NIRF signals of ASP5354 in saline- or histamine-injected tissues were significantly higher than that in normal tissues 5 min when NIRF was first detected after ASP5354 injection. In contrast, the NIRF intensity of ICG in saline- or histamine-injected tissues is similar to that in normal tissues 0.5 min when NIRF was first detected after ICG injection. These results suggested that the leakage of ASP5354 from skin capillary in saline or histamine treatment was higher than that in case using ICG compared to normal tissues. Indeed, ASP5354 is a small molecule that is not bound to plasma proteins^6^ and can, thus, readily travel through the blood vascular walls. These results can explain the fast and high elevation in NIRF intensity of ASP5354 in tumor tissues in the imaging study for KYSE850 carcinoma tissues. This might be due to the well-known effects of vascular hyperpermeability in histamine-treated and cancer tissues^26,27^. The leaked ASP5354 was collected in lymph and blood vessels and transferred in the kidneys^8^, resulting in the gradual decrease in NIRF intensity in saline or histamine injected skin and KYSE850 carcinoma tissues. ICG is non-covalently bound to plasma proteins, including albumin, which transforms them into macrosize ICG-protein complexes^1^ that do not typically leak through the blood vessel walls unless the intercellular space in the vessel walls is enough amplified by the vascular hyperpermeability effect. Although the NIRF intensity of ICG was elevated in histamine-treated skin over 90 min, the intensity in KYSE850 carcinoma tissues was similar to that in normal tissues. These results suggest that the expanded width between intercellular spaces in KYSE850 carcinoma tissues is narrower than those that are instantly expanded by the vascular hyperpermeability effect of histamine, resulting in limited leakage of ICG-protein complexes through the blood vessels in KYSE850 carcinoma tissues. In the recent study on in vivo imaging of MKN-45 human gastric cancer tissues in a cancer xenograft mouse model, normal and MKN-45 cancer-bearing mice injected intravenously with ICG demonstrated non-difference in NIRF intensity between normal and cancer tissues^14^. Therefore, vascular permeability of ICG also should be limited in.MKN-45 cancer tissues. Heptamethine indocyanine-albumin covalent complexes require a long time to accumulate in cancer tissues^28^. ICG is immediately transferred to the liver after intravenous administration, and ICG-protein noncovalent complexes should not leak through the blood vessel walls in KYSE850 carcinoma tissues immediately after ICG injection.

Fluorescence techniques for ESCC imaging have been studied using fluorescence-labeled peptides that bind specifically to ESCC cells^18–21^. The accumulation of peptide contrast agents in ESCC tissues requires as long as one day, however, unlike these peptides, ASP5354 lacks an ESCC-recognized molecular site for targeting. After intravenous injection, a larger proportion of ASP5354 immediately flowed into ESCC tissues than into normal tissues. However, ASP5354 flowed away from the tumor area without binding to the ESCC cells. Therefore, NIRF must be carefully monitored immediately after ASP5354 injection, as in MKN-45 cancer^14^. Generally, NIRF imaging systems use recording devices that can assist in confirming short-term changes in the NIRF intensity.

Using a xenograft mouse model, I demonstrated the potential of ASP5354 for ESCC imaging. However, the efficiency and proper dosage of ASP5354 for ESCC diagnosis in humans remain to be determined. Moreover, although NIRF imaging experiments were performed using a clinically available NIRF device in this study, because endoscopes are generally used for clinical ESCC diagnosis, ESCC imaging in clinical endoscopic settings using a NIRF camera system must be evaluated.

In conclusions, this study demonstrated the potential of ASP5354 for ESCC imaging using a KYSE850 human ESCC xenograft mouse model and the underlying mechanisms. After the intravenous administration of ASP5354, KYSE850 carcinoma tissues under the skin of the neck of mice were rapidly and clearly imaged using the NIRF of ASP5354 and an NIRF camera system at a high C/N ratio. The mechanisms that enabled imaging relied on the rapid and easy leakage of ASP5354 from the capillaries into the stroma of carcinoma tissues. However, the NIRF intensity of ICG did not increase in carcinoma tissues. Collectively, these findings suggest that ASP5354 is a promising imaging contrast agent for ESCC diagnosis as it can readily distinguish between ESCC and normal tissues based on distinct differences in NIRF intensity.

## Methods

### Experimental animals

All experimental protocols were approved by Institutional Animal Care and Use Committee of ITECHLAB Co., Ltd. (registration no.: ITL-21-MV-321) and Mie University Animal Ethics Committee (registration no.: MIE23-37). All methods were carried out in accordance with the guidelines issued by Science Council of Japan, Institutional Animal Care and Use Committee of ITECHLAB Co., Ltd., and Mie University Animal Ethics Committee. This study was carried out in compliance with the ARRIVE guidelines. BALB/cSlc-nu mice (male; age: 5 weeks; mean weight: 20 g) were obtained from Japan SLC, Inc. (Shizuoka, Japan). All mice were housed for 6 days under specific pathogen-free conditions at 20–25 °C before commencing the experiments, which were performed after the mice were anesthetized by subcutaneous injections of ketamine (75 mg/kg) and medetomidine (1 mg/kg). Wistar rats (male; 13-week-old; mean weight: 300 g) were obtained from Japan SLC Inc. (Shizuoka, Japan). All rats were housed under specific pathogen-free conditions at 22–24 °C prior to the experiments. Rats were anesthetized using pentobarbital sodium salt in saline.

### Materials

ASP5354, formerly designated TK-1 (C_135_H_197_N_4_O_73_Cl, molecular weight: 3079), was prepared as previously described (Suppl. Fig. 3 and Suppl. Synthesis procedure for ASP5354)^8^. The following regents were used in this study: RPMI-1640 (FUJIFILM Wako Chemicals Co., Ltd., Osaka, Japan), penicillin–streptomycin solution (5000 U/mL; Thermo Fisher Scientific K.K., Tokyo, Japan), fetal bovine serum (FBS; MP Bio Japan, Tokyo, Japan), ketamine (Daiichi Sankyo Propharma Co., Ltd., Tokyo, Japan), medetomidine (Kyoritsu Seiyaku Co., Ltd., Tokyo, Japan), pentobarbital sodium salt (TCI Japan, Tokyo, Japan), saline (Otsuka Pharmaceutical Co., Ltd., Tokyo, Japan), histamine (Wako Chemicals Co., Ltd., Osaka, Japan), and ICG (MP Biomedicals, LLC, Solon, OH, USA). A list for chemicals is in Supplementary information (Suppl. List of chemicals and instruments).

### Instruments

NIRF imaging was performed using a clinically available Photodynamic Eye camera system (PDE, Hamamatsu Photonics K.K., Shizuoka, Japan, Suppl. Fig. 4) optimized for ICG, equipped with a 760-nm light-emitting diode for excitation and a charge-coupled device for detection. An optical high-pass filter for NIRF detection was placed in front of the charge-coupled device detector. The video images were recorded using a personal computer. The measurement conditions were as follows: brightness, 6 a.u.; contrast, 5 a.u.; excitation level, 1–10 a.u.. In the in vivo investigation of carcinoma imaging, NIRF intensities at the center of the carcinoma and normal tissues (5 mm × 5 mm square) and NIRF intensities on the horizontal axis of the carcinoma and normal tissues were analyzed using a region-of-interest analysis program (Hamamatsu Photonics K.K.). PDE camera system is for direct observation by the human eye and the non-linearity is embedded in the instrument software. For in vivo imaging of the vascular permeability of ASP5354, NIRF intensities were analyzed in the regions of histamine injection sites, saline injection sites, and non-treatment sites. NIRF microscopic observation of tissue sections and KYSE850 cells was performed at 20 °C using an Axiovert 200 microscope (Carl Zeiss Co., Ltd., Oberkochen, Germany) equipped with an object lens Plan-Apochromat 20 × /0.75 (Carl Zeiss Co., Ltd.) and a monochrome camera (Axio CamMRm; Carl Zeiss Co., Ltd.). Microscopic NIRF was measured in the dark for 60 s using a 1 × 1 binning mode with the 41037 Li-Cor filter set for IR Dye 800 (excitation bandpass, 720–760 nm; emission long-pass, > 780 nm; Chroma Technology, Bellows Falls, VT, USA), and images were obtained using AxioVision 4.8 software (Carl Zeiss Co., Ltd.). An ECLIPSE E600 microscope (Nikon Corp., Tokyo, Japan) equipped with an E8400 camera (Nikon Corp.) was used to observe the sections stained with hematoxylin and eosin (H&E). A list for instruments is in Supplementary information (Suppl. List of chemicals and instruments).

### Cell lines, cell culture, and mouse model preparation

KYSE850 cells (human esophageal squamous carcinoma cells) were purchased from the JCRB Cell Bank (Osaka, Japan). Cells were cultured in RPMI-1640 medium containing 10% FBS and an antibiotic solution (penicillin and streptomycin) at 37 °C and 5% CO_2_. After culture, the cells were washed with RPMI-1640, and a cell suspension (7.5 × 10^7^ cells/mL) was prepared. Nude mice were anesthetized and a cell suspension (0.2 mL) was subcutaneously injected into the neck using a 25-G needle. RPMI-1640 (0.2 mL) was injected into the necks of healthy mice as a vehicle control. After ASP5354 is intravenously administered, it is immediately transported to the kidneys, where it emitted a strong NIRF signal^6,9^. Therefore, in this study, KYSE850 carcinoma cells were implanted under the skin on the back of the neck to reduce the interference from NIRF emissions in the kidneys. All mice were housed for 18 days, after which NIRF imaging was performed. The tumor size was 15.2 ± 2.4 mm at the long side and 11.5 ± 1.1 mm at the short side (means ± standard deviation (SD)) (*n* = 10 rats) at NIRF imaging.

### In vivo NIRF carcinoma imaging

KYSE850 carcinoma xenografts (*n* = 7 for ASP5354 and *n* = 3 for ICG) and vehicle control mice (*n* = 3 for ASP5354 and *n* = 3 for ICG) were intravenously injected with ASP5354/saline (24 μmol/L) or ICG/saline (24 μmol/L) into the tail at a dose of 120 nmol/kg body weight. Because this dose was suitable in the previous experiments for MKN-45 cancer mouse imaging using a PDE camera system^14^ and non-toxicological changes of body were observed during the imaging experiments, this dose was applied to all mice in this study. In vivo NIRF imaging of the entire back was performed in the dark using a PDE camera system at 0 (before ASP5354 or ICG injection), 0.5, 5, 10, and 30 min after ASP5354 or ICG injection. The imaging for ASP5354 was performed at moderate excitation level (4 a.u.) of camera system, for the ICG imaging moderate (4 a.u.) and high (8 a.u.) excitation levels were employed.

### NIRF imaging and histopathological analysis of tissue sections

After in vivo NIRF imaging of the back, the mice were euthanized, and the carcinoma-affected skin was excised. The tissues were frozen and sectioned, and NIRF imaging was performed using an Axiovert 200 microscope in the dark. Frozen sections of the adjacent tissues were stained with H&E and observed under a microscope as controls.

### In vitro assessment of ASP5354 uptake in KYSE850 cells

KYSE850 cells (1 × 10^5^ cells/mL) were incubated in 0.5 mL of PBS solution with 2.4 μmol/L ASP5354 at 37 °C for 10 min. The culture solution was then centrifuged (500 rpm, 20 °C, 5 min), and the obtained cells were washed five times with PBS (0.5 mL). The centrifuged cells were suspended in 0.1 mL of PBS (pH 7.4) and observed under an Axiovert 200 microscope equipped with a monochrome camera at 20 °C, and NIRF was measured.

### In vivo NIRF imaging of vascular permeability of ASP5354

The vascular permeability of ASP5354 was assessed using in vivo NIRF imaging of the back of rats. Large rats were used in the experiments so that renal fluorescence did not interfere with vascular permeability measurements in the back, and saline and histamine treatment could be evaluated on the same back. The back of rats was shaved, and saline (0.05 mL per site for 300 g rats) or histamine (4.5 mmol/L in saline, 0.05 mL per site for 300 g rats) was injected in the back dermis. Immediately, ASP5354 (250 nmol/kg body weight) or ICG (250 nmol/kg body weight) was intravenously injected into the tails under measurement of NIRF, which was measured 0.5, 5, 10, 30, 60, and 90 min after injection. In order to simultaneously measure the fluorescence intensities in normal, saline-injected, and histamine-injected tissues at the same excitation level of camera system, the excitation levels for measuring in vivo NIRF of ASP5354 and ICG were individually set.

### Statistical analysis

NIRF data are presented as mean values ± standard deviation (SD). Statistical analyses were performed using Student’s *t*-test.

## Supplementary Information

Supplementary Information.

## Data Availability

The datasets used and/or analyzed during the current study are available from the corresponding author upon reasonable request**.**
