# Supplementary material for: Ready vascular permeability of a near-infrared fluorescent agent ASP5354 for intraoperative ureteral identification enables imaging of carcinoma tissues
Source: Sci Rep. 2023 Jun 17;13:9832. doi: 10.1038/s41598-023-37025-z (PMC10276870; doi:10.1038/s41598-023-37025-z)
Supplement: Supplementary file 1 — Supplementary Information. [file 41598_2023_37025_MOESM1_ESM.pdf]

Electronic Supplementary Material

Title: Ready vascular permeability of a near-infrared fluorescent agent ASP5354 for intraoperative ureteral identification enables *in vivo* imaging of carcinoma tissues

Journal: Scientific Reports

Author: Katsunori Teranishi *

Author affiliation: Graduate School of Bioresources, Mie University, 1577 Kurimamachiya, Tsu, Mie, 514-8507, Japan

*** Corresponding author:** Katsunori Teranishi
Graduate School of Bioresources, Mie University, 1577 Kurimamachiya, Tsu, Mie 514-8507, Japan. E-mail: teranisi@bio.mie-u.ac.jp, Fax: +81-59-231-9615

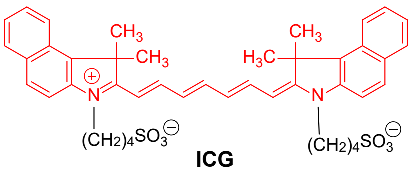

ICG

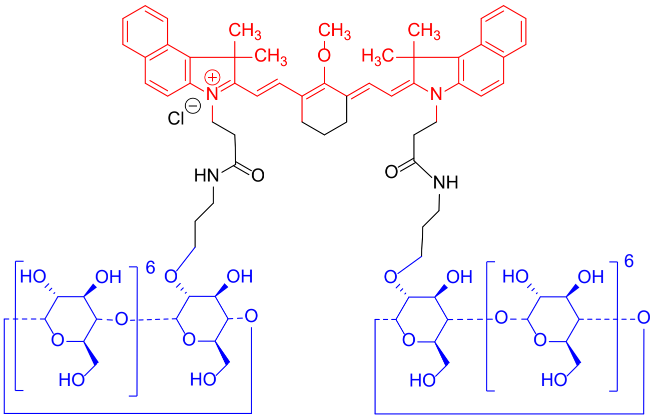

ASP5354

Suppl. Fig. 1. Chemical structures of indocyanine green (ICG) and ASP5354. Heptamethine indocyanine and *β*-cyclodextrin moieties appear red and blue, respectively.

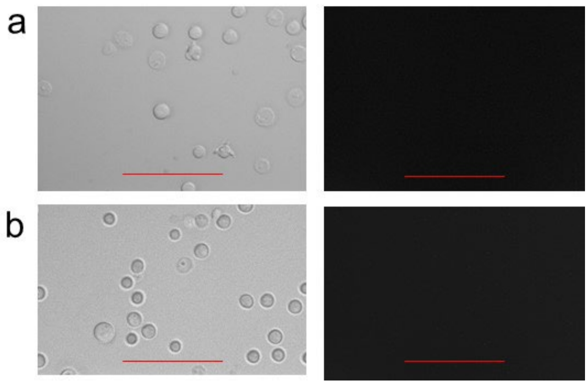

Suppl. Fig. 2. NIRF imaging of ASP5354 uptake by KYSE850 cells. Left and right photos are acquired under white light and NIRF, respectively. (**a**) Without ASP5354. (**b**) After incubation with ASP5354 (2.4 µmol/L) for 10 min. NIRF appears white. Scale bar: 100 µm. KYSE850 cells (1 × 10^5 cells/mL) were incubated in 0.5 mL of PBS solution with 2.4 µmol/L ASP5354 at 37 °C for 10 and 60 min. The culture solution was then centrifuged (500 rpm, 20 °C, 5 min), and the obtained cells were washed five times with PBS (0.5 mL). The centrifuged cells were suspended in 0.1 mL of PBS (pH 7.4) and observed under an Axiovert 200 microscope equipped with a monochrome camera at 20 °C, and NIRF was measured.

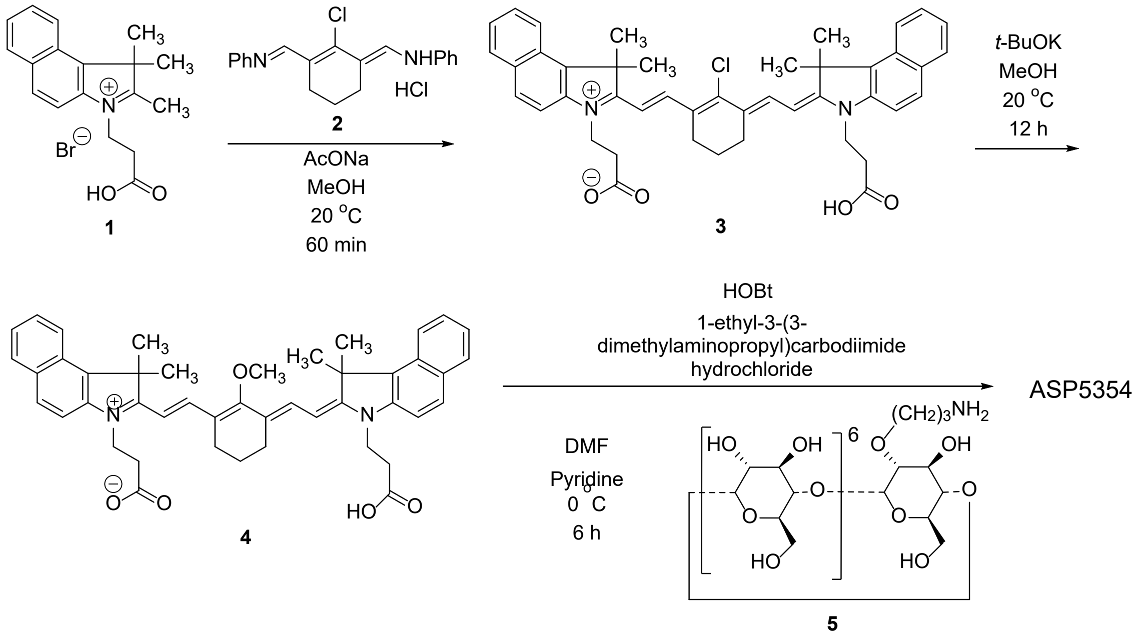

Suppl. Fig. 3. Synthesis of ASP5354.

Suppl. Synthesis procedure for ASP5354.

*1,1,2-Trimethyl[1H]-benz[e]indole-3-propanoic acid (**1**)*

1,1,2-Trimethyl[1*H*]-benz[*e*]indole-3-propanoic acid (**1**) was prepared as previously described [29]. A mixture of 1,1,2-trimethyl[1*H*]-benz[*e*]indole (1.6 g, 7.66 mmol) and 3-bromopropionic acid (1.28 g, 8.42 mmol) in 1,2-dichlorobenzen (16 mL) was heated at 120ºC for 24 h. After cooling, toluene (16 mL) was added and 1 h thereafter the resulting crystals was filtered, washed with toluene and dichloromethane, and dried to afford **1** (1.52 g, 55% yield).

*3-(2-carboxyethyl)-2-[2-[3-[2-[3-(2-carboxyethyl)-1,3-dihydro-1,1-dimethyl-2H-benz[e]indol-2-ylidene]ethylidene]-2-chloro-1-cyclohexen-1-yl]ethenyl]-1,1-dimethyl-1H-benz[e]indolium,inner salt (**3**)*

A mixture of **1** (2.25 g, 6.23 mmol), *N*-[(3-(anilinomethylene)-2-chloro-1-cyclohexen-1-yl)methylene]-aniline monohydrochloride (**2**) (1.05 g, 2.92 mmol), and sodium acetate (2.5 g, 30 mmol) in methanol (50 mL) was stirred at 20ºC for 60 min. After 1.0 mM aqueous HCl (20 mL) and water (400 mL) were added to the resulting mixture, the precipitate was collected by filtration, followed by sequential washing with 1.0 mM aqueous HCl (100 mL), water (700 mL), ethyl acetate (200 mL), a mixture of ethyl acetate (25 mL) and acetone (25 mL), and ethyl acetate (400 mL), after which it was dried under vacuum to afford **3** (1.70 g, 83% yield). The purity of **3** was checked by HPLC-PDA-MS analysis, and **3** was used without further purification.

*3-(2-carboxyethyl)-2-[2-[3-[2-[3-(2-carboxyethyl)-1,3-dihydro-1,1-dimethyl-2H-benz[e]indol-2-ylidene]ethylidene]-2-methoxy-1-cyclohexen-1-yl]ethenyl]-1,1-dimethyl-1H-benz[e]indolium (**4**)*

To a mixture of **3** (0.17 g, 0.243 mmol) and anhydrous methanol (5.0 mL) added *t*-

BuOK (0.3 g, 2.68 mmol) at 20ºC, and the resulting reaction mixture was protected from light and stirred at 20ºC for 12 h. The reaction was checked for completion by HPLC-PDA-MS. Subsequently, 1 mM aqueous HCl (3.0 mL) was added, and the resulting mixture was added to water (50 mL). After the mixture was evaporated under reduced pressure to remove the methanol, the precipitate was collected by filtration, followed by washing with water, and dried under vacuum to afford **4** (0.17 g, 100% yield), as a green solid. The purity of **4** was checked by HPLC-PDA-MS and ^{1}H NMR analyses, and **4** was used without further purification because of its excellent purity. ^{1}H NMR (500 MHz, DMSO-d_6, 27ºC, DMSO: 2.49 ppm): δ 1.8 (2H, m), 1.86 (12H, s), 2.57 (4H, m), 2.62 (4H, t, J = 6.7 Hz), 4.43 (4H, t, J = 6.7 Hz), 6.29 (2H, d, J = 14.0 Hz), 7.44 (2H, t, J = 8.5 Hz), 7.60 (2H, t, J = 8.5 Hz), 7.69 (2H, d, J = 8.5 Hz), 7.98 (2H, d, J = 8.5 Hz), 8.01 (2H, d, J = 8.5 Hz), 8.03 (2H, d, J = 14.0 Hz), 8.20 (2H, d, J = 8.5 Hz). HR-MS (ESI, positive mode): m/z calculated for $[C_{45}H_{46}N_2O_5 + H]^+$ 695.3479; found: 695.3455 $[M + H]^+$.

*2A-O-(3-Aminopropyl)-β-cyclodextrin (**5**)*

2A-*O*-(3-Aminopropyl)-*β*-cyclodextrin (**5**) was prepared as previously described [30]. *β*-Cyclodextrin (5 g, 4.4 mmol) was dissolved in DMSO (50 mL) and to the solution was added molecular sieves 4A (5 g). The mixture was then stirred at 20ºC for about 12 h and 60% NaH (0.53 g, 13.2 mmol) was added, and the resulting mixture was stirred for 9 h. *N*-(3-bromopropyl)phthalimide (3.54 g, 13.2 mmol) was added to the mixture and stirred at 20ºC for 20 h. The molecular sieves were removed by filtration and the filtrate was poured into acetone (750 mL) with stirring, and the precipitates formed were allowed to settle at 0ºC for 1 h. The precipitates were filtered and washed with acetone and dried in the vacuum to give the crude product, which involved unreacted *β*-cyclodextrin and 2A-*O*-(3-phthalimidopropyl)cyclomaltoheptaose. The crude product was dissolved in warm water, and placed on a Fuji Silysia Chromatorex-ODS DM1020T gel column (40 ϕ x 100 mm) and eluted with water to 30% aqueous MeOH. The pure fractions of 2A-*O*-(3-phthalimidopropyl)cyclomaltoheptaose were combined and then concentrated under reduced pressure to give a solid (1.7 g, 29% yield). A portion was crystallized in a mixture of H_2O and MeOH: colorless prisms, mp 235ºC (decomp), IR (KBr): ν_{max} = 3405, 2930, 1765, 1715, and 1632 cm^{-1}; UV (H_2O, 25ºC): λ_{max} = 299 nm (*ε 1710*) and 221 nm (*ε 32,500*); ^{1}H NMR (500 MHz, DMSO-d_6, 25ºC, TMS: 0.00 ppm): δ 1.85 (2H, m, $NCH_2CH_2CH_2O$), 3.23 (1H, dd, J = 3.1 and 9.8 Hz, H-2 of glucose unit with phtalimidopropyl group), 3.3–3.8 (majority, m, H of cyclomaltoheptaose unit and $NCH_2CH_2CH_2O$), 3.86 (1H, m, NCHCHC*H*O), 3.84 (1H, t, J = 9.8 Hz, H-3 of glucose unit with phtalimidopropyl group), 4.80–4.85 (6H, m, H-1 of glucose unit without phtalimidopropyl group), 5.00 (1H, d, J = 3.1 Hz, H-1 of glucose unit with phtalimidopropyl group), and 7.8–7.9 (4H, m, ArH); ^{13}C NMR (DMSO-d_6, 25ºC, TMS: 0.00 ppm): δ28.72 ($NCH_2CH_2CH_2O$), 34.75 ($NCH_2CH_2CH_2O$), 60.1 (C-6 of glucose unit with and without phtalimidopropyl group), 69.98 ($NCH_2CH_2CH_2O$), 71.9–73.3 (C-2, 3, 5 of glucose unit), 81.07 (C-2 of glucose unit with phtalimidopropyl group), 82.7–82.6 (C-4 of glucose unit), 100.75 (C-1 of glucose unit with phtalimidopropyl group), 102.0–102.4 (C-1 of glucose unit without phtalimidopropyl group), 123.37 (Ar), 131.91 (Ar), 134.75 (Ar), and 168.46 (*C*(O)N); MS m/z : 1322.8 (M+1); *Anal.* Found: C, 46.98; H, 5.89; N, 1.00%, calcd for $C_{53}H_{79}O_{37}N + 2H_2O$: C, 47.01; H, 5.88; N, 1.03%.

To a mixture of MeOH (33 mL) and DMSO (17 mL) was added 2A-*O*-(3-phthalimidopropyl)cyclomaltoheptaose (3.4 g, 2.57 mmol) and hydrazine monohydrate

(0.64 g, 12.9 mmol), and then the mixture was stirred at 50ºC for 1 day. The reaction solution was poured into acetone (750 mL) with stirring, and the precipitates were filtered and washed with acetone. The crude product was dissolved in water and purified using a Sephadex CM-25 gel column, eluted with water to 0.5 N aqueous NH_3. The pure fractions of **5** were combined and then concentrated under reduced pressure to give a solid (2.54 g, 83% yield): colorless powder, mp 263ºC (decomp); IR (KBr): ν_{max} = 3400, 2920, and 1634 cm^{-1}; ^{1}H NMR (500 MHz, DMSO-d_6, 40ºC, TMS: 0.00 ppm): δ 1.57 (2H, m, $NCH_2CH_2CH_2O$), 2.57 (2H, m, $NCH_2CH_2CH_2O$), 3.21 (1H, dd, J = 3.1 and 9.8 Hz, H-2 of glucose unit with 3-aminopropyl group), 3.2–3.8 (majority, m, H of cyclomaltohexaose unit and $NCH_2CH_2CH_2O$), 4.7–4.8 (6H, m, H-1 of glucose unit without 3-aminopropyl group), and 4.95 (1H, d, J = 3.1 Hz, H-1 of glucose unit with 3-aminopropyl group); ^{13}C NMR (DMSO-d_6, 40ºC, TMS: 0.00 ppm): δ 32.28 ($NCH_2CH_2CH_2O$), 37.81 ($NCH_2CH_2CH_2O$), 59.6–60.1 (C-6 of glucose unit with and without 3-aminopropyl group), 69.70 ($NCH_2CH_2CH_2O$), 71.73–73.00 (C-2, 3, 5 of glucose unit), 80.51 (C-2 of glucose unit with 3-aminopropyl group), 81.20–82.13 (C-4 of glucose unit), 100.24 (C-1 of glucose unit with 3-aminopropyl group), and 101.74–102.00 (C-1 of glucose unit without 3-aminopropyl group); MS *m/z* : 1192.8 (M+1); *Anal.* Found: C, 41.89; H, 6.19; N, 1.06%, calcd for $C_{45}H_{77}O_{35}N + H_2O$: C, 41.91; H, 6.17; N, 1.09%.

ASP5354

A mixture of **4** (60 mg, 0.086 mmol), **5** (0.26 g, 0.218 mmol), HOBt (34 mg, 0.22 mmol), and 1-ethyl-3-(3-dimethylaminopropyl)carbodiimide hydrochloride (74 mg, 0.39 mmol) in DMF (0.6 mL) and pyridine (1.2 mL) was stirred at 0ºC for 6 h in the dark. The reaction was checked for completion by HPLC-PDA-MS. Acetone (10 mL) was added to the resulting mixture, and the precipitate was collected by filtration, washed with acetone, and dried under vacuum. The solid obtained was dissolved in 0.1% aqueous TFA and purified by C-18 open column chromatography, using 1 mM aqueous HCl and 1 mM HCl/methanol as elution solvents. The product-containing fractions were evaporated under reduced pressure to remove almost all solvent, without complete evaporation of the solvent (to avoid decomposition of the product), after which acetone was added to the resulting residue to powder the product. The precipitate was collected by filtration, followed by washing with acetone, and dried under vacuum to afford ASP5354 as a green powder. The resulting powder was dissolved followed filtration with a 0.2-μm membrane filter, and the filtrate was freeze-dried in the dark to afford ASP5354 as a green amorphous powder (0.18 g, 69%). ^{1}H NMR (500 MHz, D_2O, 40ºC, acetone: 2.26 ppm): δ 1.54 (2H, m), 1.68 (2H, m), 1.98 (2H, m), 2.19 (6H, s), 2.20 (2H, m), 2.30 (6H, s), 2.6-2.85 (10H, m), 2.95 (2H, m), 3.00 (4H, m), 3.08 (2H, t, J = 12 Hz), 3.17 (2H, dd, J = 3.7, 9.8 Hz), 3.26 (2H, t, J = 9.8 Hz), 3.35–4.30 (m), 4.35 (2H, t, J = 9.2 Hz), 4.50 (2H, t, J = 9.2 H), 4.52 (2H, m), 4.63 (2H, m), 4.87 (2H, d, J = 3.7 Hz), 4.95 (d, J = 3.1 Hz), 4.97 (2H, d, J = 3.7 Hz), 5.08 (2H, d, J = 3.7 Hz), 5.15 (2H, d, J = 4.3 Hz), 5.25 (2H, d, J = 3.7 Hz), 5.29 (2H, d, J = 3.7 Hz), 6.30 (2H, d, J = 14.6 Hz), 7.58 (4H, m), 7.73 (2H, d, J = 8.5 Hz), 7.95 (2H, m), 8.25 (2H, m), 8.32 (2H, d, J = 14.6 Hz), 8.35 (2H, d, J = 8.5 Hz). UV-visible-NIR (EtOH, 25ºC): λ_{max} = 800 nm (ε *200000*); UV-visible-NIR (PBS, pH 7.4, 25ºC): λ_{max} = 800 nm (ε *200000*); UV-visible-NIR (DMSO, 25ºC): λ_{max} = 809 nm (ε *180000*); Φ_F (EtOH, 25ºC): 0.03; Φ_F (PBS, pH 7.4, 25ºC): 0.048; Φ_F (DMSO, 25ºC): 0.12; fluorescence (EtOH, 25ºC): λ_{max} = 811 nm; fluorescence (PBS, pH 7.4, 25ºC): λ_{max} = 812 nm; fluorescence (DMSO, 25ºC): λ_{max} = 825 nm; MS (ESI, positive mode): *m/z*

calculated for $[C_{135}H_{197}N_4O_{73}]^+$ 3042, found: 3042 $[M]^+$.

References

29. Ye, Y. *et al.*, Multivalent carbocyanine molecular probes: synthesis and applications. *Bioconjug. Chem.* **16**(1), 51–61 (2005).
30. Teranishi, K. & Tanabe, S. Regiospecific alkylation dependent on alkyl chain length of N-bromoalkylphthalimides and an efficient preparation of 2-O-aminoalkyl cyclomaltooligosaccharides (cyclodextrins). *ITE Lett.* **1**, 53–60 (2000).

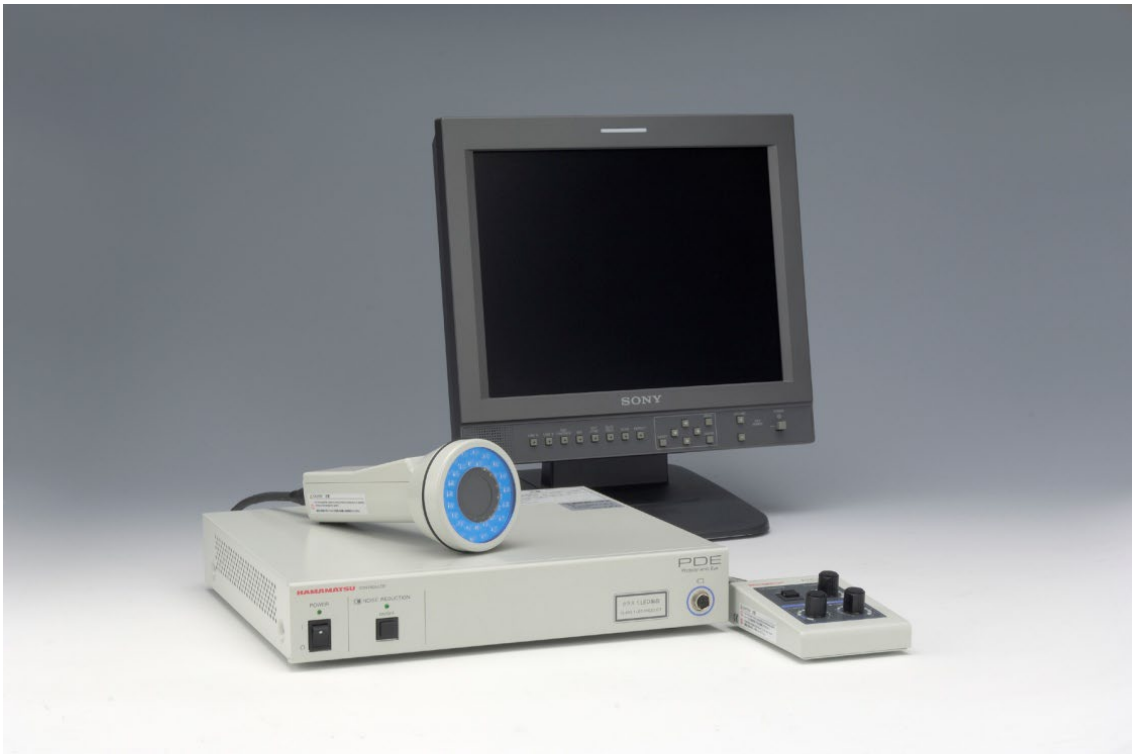

Suppl. Fig. 4. Photodynamic Eye camera system

Suppl. List of chemicals and instruments

Chemicals

Name	Cord no.	Company
acetone	016-00346	Wako Chemicals Co., Ltd.
2A-*O*-(3-aminopropyl)-*β*-cyclodextrin		synthesized as previously described [30]
aqueous HCl	083-03435	Wako Chemicals Co., Ltd.
aqueous NH_3	016-03146	Wako Chemicals Co., Ltd.
ASP5354		synthesized as previously described [8]
3-bromopropionic acid	028-12872	Wako Chemicals Co., Ltd.
β-cyclodextrin	034-08345	Wako Chemicals Co., Ltd.
1,2-dichlorobenzen	042-07786	Wako Chemicals Co., Ltd.
D_2O	151882	Sigma-Aldrich
dichloromethane	135-02441	Wako Chemicals Co., Ltd.
DMSO	043-07211	Wako Chemicals Co., Ltd.
DMSO-d_6	439037-25G	Sigma-Aldrich
1-ethyl-3-(3-dimethylaminopropyl)carbodiimide hydrochloride	348-03631	Wako Chemicals Co., Ltd.
ethyl acetate	057-00353	Wako Chemicals Co., Ltd.
fetal bovine serum	2916554	MP Bio Japan
60% NaH	191-07662	Wako Chemicals Co., Ltd.
histamine	088-00641	Wako Chemicals Co., Ltd.
hydrazine monohydrate	081-00893	Wako Chemicals Co., Ltd.
1-hydroxy-1H-benzotriazole monohydrate (HOBt)	349-03622	Wako Chemicals Co., Ltd.
indocyanin green (ICG)	155020	MP Biomedicals
ketamine	1119400A2038	Daiichi Sankyo Propharma Co., Ltd.
medetomidine	DOL04-AS2101	Kyoritsu Seiyaku Co., Ltd.
methanol	133-16771	Wako Chemicals Co., Ltd.
molecular sieves 4A	04168-65	Nacalai Tesque Inc.
N-(3-bromopropyl)phthalimide	022-12532	Wako Chemicals Co., Ltd.
N,N-dimethylformamide (DMF)	045-02916	Wako Chemicals Co., Ltd.
N-[(3-(anilinomethylene)-2-chloro-1-cyclohexen-1-yl)methylene]-aniline monohydrochloride	407232-5G	Sigma-Aldrich

penicillin–streptomycin solution	15070063	Thermo Fisher Scientific K.K.
pentobarbital sodium salt	P0776	TCI Japan,
phosphate-buffered saline (PBS)	168-27155	Wako Chemicals Co., Ltd.
potassium tert-butoxide (*t*-BuOK)	169-08422	Wako Chemicals Co., Ltd.
pyridine	162-05313	Wako Chemicals Co., Ltd.
RPMI-1640	189-02025	FUJIFILM Wako Chemicals Co., Ltd.
saline	1325	Otsuka Pharmaceutical Co., Ltd.
sodium acetate	198-01072	Wako Chemicals Co., Ltd.
toluene	204-01866	Wako Chemicals Co., Ltd.
trifluoroacetic acid (TFA)	204-02743	Wako Chemicals Co., Ltd.
1,1,2-trimethyl[1*H*]-benz[*e*]indole	323-29461	Wako Chemicals Co., Ltd.
1,1,2-trimethyl[1H]-benz[*e*]indole-3-propanoic acid		synthesized as previously described [29]

Instruments

Name	Cord no.	Company
fluorescence microscope	Axiovert 200	Carl Zeiss Co., Ltd.
HPLC system	Gulliver	JASCO
infrared spectrometer	FT/IR-410	JASCO
mass spectrometer	ZQ 4000	Waters Corporation
mass spectrometer	Orbitrap Velos ETD	Thermo Fisher Scientific
microscope	ECLIPSE E600	Nikon Corp.
NIRF camera system	Photodynamic Eye C9830	Hamamatsu Photonics K.K.
NMR spectrometer	JNM-A500	JEOL
spectrofluorometer	FP-6600	JASCO
UV/VIS spectrophotometer	V-530	JASCO
